# Analysing the preferences for family doctor contract services in rural China: a study using a discrete choice experiment

**DOI:** 10.1186/s12875-020-01223-9

**Published:** 2020-07-25

**Authors:** Peipei Fu, Yi Wang, Shimeng Liu, Jiajia Li, Qiufeng Gao, Chengchao Zhou, Qingyue Meng, Sean Sylvia

**Affiliations:** 1 NHC Key Laboratory of Health Economics and Policy Research, School of Health Care Management, Shandong University, Jinan, 250012 China; 2 School of Public Health, Shandong University, Jinan, 250012 China; 3 Key Lab of Health Technology Assessment, National Health Comission, School of Public Health, Fudan University, Shanghai, China; 4 Center for Experimental Economics in Education, Shaanxi Normal University, Xi’an, China; 5 Key Lab of Health Economics and Policy Research, School of Public Health, Shandong University, 44 Wen-hua-xi Road, Jinan, 250012 Shandong China; 6 China Center for Health Development Studies, Peking University, No. 38 Xueyuan Road, Haidian District, Beijing, 100191 China; 7 Department of Health Policy and Management, Gillings School of Global Public Health, University of North Carolina at Chapel Hill, Chapel Hill, North Carolina USA

**Keywords:** Primary care, Family doctor contract services, Rural resident preferences, China

## Abstract

**Background:**

Preliminary evaluations have found that family doctor contract services (FDCSs) have significantly controlled medical expenses, better managed chronic diseases, and increased patient satisfaction and service compliance. In 2016, China proposed the establishment of a family doctor system to carry out contract services, but studies have found the uptake and utilization of these services to be limited. This study aimed to investigate rural residents’ preferences for FDCSs from the perspective of the Chinese public.

**Methods:**

A discrete choice experiment (DCE) was performed to elicit the preferences for FDCSs among rural residents in China. Attributes and levels were established based on a literature review and qualitative methods. Five attributes, i.e., cost, medicine availability, the reimbursement rate, family doctor competence, and family doctor attitude, were evaluated using a mixed logit model.

**Results:**

A total of 609 residents were included in the main DCE analysis. The respondents valued the high competence (coefficient 2.44, [SE 0.13]) and the good attitude (coefficient 1.42, [SE 0.09]) of family doctors the most. Cost was negatively valued (coefficient − 0.01, [SE 0.01]), as expected. Preference heterogeneity analysis was conducted after adjusting the interaction terms, and we found that rural residents with higher educational attainment prefer a good attitude more than their counterparts with lower educational attainment. The estimated willingness to pay (WTP) for “high” relative to “low” competence was 441.13 RMB/year, and the WTP for a provider with a “good” attitude relative to a “poor” attitude was 255.77 RMB/year.

**Conclusion:**

The present study suggests that strengthening and improving the quality of primary health care, including the competence and attitudes of family doctors, should be prioritized to increase the uptake of FDCSs. The contract service package, including the annual cost, the insurance reimbursement rate and individualized services, should be redesigned to be congruent with residents having different health statuses and their stated preferences.

## Background

Foundational for population health, expanding access to quality primary care services is a key priority for all public health systems [1, 2]. In developed countries, family doctors are at the forefront of primary care delivery and play an indispensable role in providing comprehensive primary health services [3, 4]. While the terms used to describe family doctors—such as general practitioner (GP) and family physician—vary internationally, in this study, we define family doctor as a physician, nurse or public physician having specialist training in primary care who provides family doctor contract services.

The evidence previously gathered in a variety of countries has emphasized the role of primary care in achieving better population health outcomes while reducing costs. Indeed, physicians and public health systems providing more coordinated services have consistently achieved better health outcomes [3, 5]. Moreover, primary health care provided by family doctors has been shown to be highly cost-effective, preventing potential health complications and the need for specialized care through early prevention and screening [6, 7]. Preliminary evaluations have found that family doctor contract services have significantly controlled medical expenses, better managed chronic diseases, and increased patient satisfaction and service compliance [8–10]. Family doctors often play the role of a “gatekeeper” in the primary health care system, ideally promoting the efficient allocation of health resources and preventing the excessive medical costs associated with more expensive procedures [8, 11].

To provide comprehensive, coordinated, and preventative public health care to all citizens, in 2016, the Chinese government proposed the establishment of a family doctor system to carry out contract services [12]. Family doctor contract services (FDCSs) are provided by a care team that usually consists of general practitioners, nurses, and public health physicians. The FDCSs are in its early stage, and in family doctor teams, general practitioners play the most important role. Most existing research has indicated that general practitioners in rural areas in China provide more than 95% of primary care and play a key role as the primary health gatekeeper [13]. General practitioners are contracted to provide basic medical care services, public health services, and individualized health management. Under the working principles of full notification, a voluntarily signed contract and standardized service, general practitioners establish a long-term and stable service relationship with the families whom they serve through a signed contract. The National Medical Reform Office stipulates a full coverage rate by 2020.

To improve FDCS implementation, it is important to understand residents’ willingness to sign contracts with family doctors and to explore the determinants of this willingness. However, few studies reflect the views and preferences of the public regarding FDCSs in China. In recent years, research in China has mainly focused on the significance of establishing the family doctor system [14], and most research has been in urban areas [14–17]. There are also some studies exploring the effect of the family doctor policy [8, 18, 19]. Some researchers have noted the importance of establishing stable relationships with family doctors [20], but little is known about the determinants underlining this scheme, especially among rural residents with different health statuses. In terms of methods, logistic regression has mainly been used in research on family doctors in China to seek influencing factors. The existing international literature suggests that perceived quality of care [21–23] can be an influential determinant of demand for family doctor services alongside out-of-pocket costs and insurance coverage [24, 25]. Moreover, demand varies with the socioeconomic characteristics and health status of patients [21–23]. A better understanding of the determinants of demand for FDCS contracts, particularly the attributes of the care provided in addition to the pricing structure, is critically important for designing and refining family doctor services as China continues to invest in its primary healthcare system. The current study seeks to address this gap by using a discrete choice experiment to explore residents’ preferences for FDCSs to provide information that is useful in designing and implementing the next stage of the family doctor service scheme.

## Methods

A discrete choice experiment (DCE) is a quantitative method that aims to elicit stated preferences. This method draws on Lancaster’s consumer theory, which assumes that health care interventions and policies are combinations of attributes and that individuals’ choice of these goods is based on various levels of these attributes [26]. The DCE model has been widely used to predict the probability of uptake of various contract service plans and to determine the preferences for goods services in lieu of observations of real-world market interactions.

### DCE questionnaire design

To select representative attributes that can clearly depict and capture residents’ preferences for family doctor teams under FDCSs, we developed a DCE questionnaire through qualitative methods, including a literature review and interviews with key informants. We first reviewed the international and domestic literature on primary health care providers and patients’ choices of doctors to identify which attributes were highly relevant to our study. A pilot study recruited 3 rural residents who signed an FDCS contract, 2 village doctors and 2 township hospital managers from Zhangqiu County, located in central Shandong Province, to encourage them to share their views on 1) how the family doctor service mechanism has been implemented; 2) the influencing factors considered by residents to sign family doctor contract service contracts; 3) the hindering factors that deter awareness and acceptance of this service scheme; and 4) policy recommendations to increase the family doctor signing rate. Using semi-structured interviews, we collected data about what factors influence rural residents the most when they sign family doctor contracts. A DCE workshop with 2 DCE experts was also conducted on March 23–25, 2018. The DCE experts provided valuable suggestions on describing the attributes, determining the levels for each attribute, and designing the experiment. Combined with the literature review results and the common suggestions raised by FGD participants, five determinants that impact rural residents’ decision making the most were selected.

The five attributes of FDCS contracts described below were determined to be the most relevant to uptake in our setting. A full description of the attribute selection and questionnaire implementation process is available in the Additional file 1.

1. Contract price: This attribute refers to the annual signing expenses incurred by an individual resident. After we reviewed public policies and guidelines on FDCSs enacted by central and local governments, three levels were specified for this attribute: 0 CNY, 100 CNY and 200 CNY per year [27, 28].
2. Availability of medicines: Medicine availability refers to the ability to obtain affordable medicines that are necessary for a person to maintain his or her health [29]. We selected this attribute to indicate the accessibility of health services provided by the contracted family doctor. We divided this attribute into two levels in our questionnaire: shortage and sufficient.
3. Insurance reimbursement rate: While health insurance was recently universalized in China, insurance reimbursement rates vary by plan and scheme. Previous studies have shown a close relationship between medical insurance and patients’ choice of medical treatment [30]. Referring to the reimbursement guidelines issued by the Shandong Health Commission, we divided this attribute into three levels in our questionnaire: standard reimbursement, 5% more than the standard reimbursement rate, and 10% more than the standard reimbursement rate.
4. Family doctor competence. The competence and skill of physicians are considered of great importance to patients [23, 31, 32]. This attribute refers to a resident’s attention to physician credentials and perceived competence when selecting a family doctor. We divided this attribute into three levels in our questionnaire: low, medium and high.
5. Family doctor attitude. Many studies have shown a correlation between doctors’ attitudes and patients’ medical behaviours [33–35]. Thus, we sought to investigate the relative importance of perceived attitude in the decision to sign a family doctor team. In this research, this attribute was divided into three levels: poor, normal and good.

A consistency test was performed to ensure that each respondent made realistic trade-offs and to check validity of this research. In this study, one repeated choice set question was added to each version of the questionnaire to check the preference consistency of each respondent. We excluded the information of respondents who failed the consistency test.

### Data collection

This study was conducted in Shandong Province, the second largest province in China. Within Shandong, 3 cities—Binzhou, Zibo, and Liaocheng, located in the northeast, central, and west regions of the province, respectively—were selected as study sites. Multi-stage random sampling was used to choose a sample of respondents representative of the rural residents in each selected city. To do so, 2 counties in each city were first chosen at random. Within each county, 5 townships (the administrative level below the county) and 24 households in each township were randomly chosen. In this study, the questionnaire was administered to 720 residents 18 years of age and above, which is more than the 600 observations recommended as sufficient for preference heterogeneity analysis [36]. Among the 720 questionnaires, 20 of them were incomplete, and these ineligible surveys were dropped. A total of 91 surveys failed to pass the consistency test in the questionnaire and were excluded. Finally, 609 questionnaires were included in the statistical analysis.

Data were collected in this study through a DCE questionnaire administered by teams of trained enumerators at study households. Since most respondents had low levels of educational attainment, a face-to-face interview method was applied to ensure that each respondent clearly understood the entire survey. At the beginning of each interview, the enumerators described the purpose of the study and sought participant consent. Following consent, a brief introduction to FDCSs, the recent public health policies implemented by the government, and the attributes in each choice set were explained. This explanation was then followed by a one-page introduction to the task with a warm-up choice question to check whether the respondent could fully understand the questionnaire and make trade-offs in each pair-wise choice set. Each participant was asked to imagine different hypothetical scenarios in which different family doctor contract service plans are enrolled in to enhance their health status. They were then asked to make discrete choices between 10 pair-wise combinations of scenarios. On average, it took approximately 50 min to complete the whole questionnaire, and the survey was returned to the interviewer immediately. A sample questionnaire choice is shown in Table 1.

**Table 1:** An example of a DCE question

Attributes	Contract plan 1	Contract plan 2
Cost of the contract	200 CNY/year	100 CNY/year
Availability of medicine	Easy	Difficult
Reimbursement rate	Standard	10% more
Family doctor competence	Medium	Low
Family doctor attitude	Good	Normal
**Which contract plan would you choose?**	□	□

Pleases consider that you are going to enrol in a family doctor contract service for yourself. Of the two contract plans above, which contract plan would you choose?

### Statistical analysis

The data were first double-entered and coded using EpiData version 3.1, and the final data were then transferred to STATA 14.2 for all statistical analyses.

Random utility theory provided the theoretical foundation for the analysis of the DCE data [37]. Mixed logit models were used to estimate the utility of enrolling in one contract plan [37]. We assumed that the respondents were relatively homogenous in terms of the demographic measures; hence, their preferences would be associated with choice variables. The utility function is specified as follows:

$$ {U}_i=\alpha +{\beta}_1 cost+{\beta}_2 medicin{e}_{-} sufficient+{\beta}_3 reimbursemen\_5\% more+{\beta}_4 reimbursemen{t}_{-}10\% more+{\beta}_5 competence\_ medium+{\beta}_6 competence\_\mathrm{high}+{\beta}_7 attitud{e}_{-} normal+{\beta}_8 attitud{e}_{-} good $$

All attributes were dummy coded except for the cost of the contract, which was specified as a continuous variable to facilitate the calculation of willingness to pay (WTP). WTP was calculated to measure the trade-offs among various contract attributes. WTP was estimated as the ratio of the coefficient to the negative coefficient on the contract cost attribute. The coefficients indicated the relative importance of the worst values for the categorical variables.

## Results

### Respondent characteristics

A total of 609 rural residents from 3 cities in Shandong Province were selected in the final sample for analysis after excluding 91 respondents who failed to pass the consistency test. The characteristics of the respondents are reported in Table 2. The respondents were 18–88 years of age (mean = 51.21 years old), and approximately half (52.38%) were female. A total of 20.03% of the respondents had completed high school or above, and 54.70% reported that their annual household income was less than 40,000 RMB ($5960.19 based on the exchange rate of 6.71). The percentage of participants whose household included pregnant women or children younger than 6 years of age in the past 6 months were 15.93 and 27.91%, respectively. A total of 32.40% of the respondents had chronic diseases.

**Table 2:** Demographic characteristics of 609 respondents in China

Characteristics	***N*** = 609	%
**Age, years Mean ± SD**	51.21	±13.05
**Gender**		
Female	290	47.62%
Male	319	52.38%
**Education**		
Primary school or below	209	34.32%
Middle school	278	45.65%
High school or above	122	20.03%
**Marital status**		
Married	571	93.76%
Unmarried	38	6.24%
**Household income per year**^**a**^		
< 20,000 CNY	161	26.44%
20,000–40,000 CNY	172	28.24%
40,000–70,000 CNY	175	28.74%
> 70,000 CNY	101	16.58%
**With chronic diseases**		
Yes	197	32.35%
No	412	67.65%
**With pregnant women**		
Yes	97	15.93%
No	512	84.07%
**With children aged 0–6**		
Yes	170	27.91%
No	439	72.09%
**Region**		
Zibo	210	34.48%
Liaocheng	197	32.35%
Binzhou	202	33.17%

**Source** Analysis data from the questionnaires of rural residents in China. **Notes** The average exchange rate between US$ and CNY in 2018 was US$1 = CNY6.71

### Model estimation

Table 3 shows the main effects of each attribute on utility in the mixed logistic models. The results indicate that on average, residents prefer an FDCS contract with lower costs, a higher reimbursement rate, a sufficient availability of essential drugs, and a highly competent family doctor who has a better attitude. All attributes were statistically significant at the 1% level. We want to emphasize that the size of the coefficients indicated that “family doctor competence” and “family doctor attitude” were the most valued attributes.

**Table 3:** Main effect model estimation and residents’ willingness to pay for different attributes of contract services

Attributes	Coefficient^**a**^	WTP^**b**^ (CNY)
	(SE)	(95% CI)
**Contract costs**	−0.01***	–
	(0.01)	–
**Availability of medicine (easy)**	0.63***	114.14
	(0.06)	(87.55 ~ 140.74)
**Reimbursement rate 5% more**	0.31***	56.02
	(0.06)	(33.24 ~ 78.81)
**Reimbursement rate 10% more**	0.45***	81.66
	(0.08)	(53.19 ~ 110.14)
**Medium level of family doctor competence**	1.24***	224.73
	(0.08)	(188.37 ~ 261.10)
**High level of family doctor competence**	2.44***	441.13
	(0.13)	(377.92 ~ 504.35)
**Normal family doctor attitude**	1.01***	182.18
	(0.08)	(148.04 ~ 216.33)
**Good family doctor attitude**	1.42***	255.77
	(0.09)	(212.22 ~ 299.34)

**Source** Analysis data from the questionnaires of rural residents in China. **Notes** WTP is estimated by calculating the ratios of the coefficients between each attribute level and the contract cost attribute. ^a^The coefficients represent the mean relative utility of each attribute conditional on other attributes in a choice set, and a larger coefficient means a more preferred attribute. ^b^The average exchange rate between US$ and CNY in 2018 was US$1 = CNY6.71. 95% CI = 95% confidence interval, SE = standard error, *** *p* < 0.01, ** *p* < 0.05, * *p* < 0.1

We also estimated WTP, which measures the amount that an individual would give up to improve an attribute. The WTP estimates derived from the logit model indicate that the respondents would pay 441.13 CNY for a family doctor with high (relative to low) competence in regard to diagnosis and treatment, 255.77 CNY for a family doctor with a warm and friendly (relative to bad) attitude, 114.14 CNY to have sufficient access to essential drugs, and 81.66 CNY to obtain an insurance reimbursement rate that is 10.00% higher than the standard. Thus, the competence and attitude of doctors had the highest impact on rural residents’ demand for FDCS contracts.

The standard deviation (SD) results suggested that preference heterogeneity existed among the respondents. Preference heterogeneity analysis was conducted with the interaction terms of gender, age, educational attainment and health status (whether the respondents have chronic diseases), and the results are shown in the Additional file 1. For gender, the reference group was the female group. In terms of education, the reference group was residents with a primary school level of educational attainment or below. The results shown in Table 4 indicate that individuals with higher educational attainment value a better attitude compared with those with lower educational attainment. Younger residents value the easy availability of medicine more than older people. Additionally, the availability of medicines is valued more highly by residents with a medium educational level compared to residents with a lower educational level. There were no statistically significant attribute interactions with gender and health status, indicating no preference heterogeneity among these two demographic characteristics.

**Table 4:** Model estimation of the interaction effects with different attributes and education

Attributes and levels	Coefficient	SE
Contract costs	−0.01***	0.01
Availability of medicine (easy)	0.47***	0.11
Reimbursement rate 5% more	0.27***	0.10
Reimbursement rate 10% more	0.42***	0.12
Medium competence	1.33***	0.12
High competence	2.32***	0.18
Normal attitude	0.81***	0.13
Good attitude	1.16***	0.13
Contract cost*Education_m	0.01	0.01
Contract cost*Education_h	0.01	0.01
Availability of medicine (easy)* Education_m	0.34***	0.14
Availability of medicine (easy)* Education_h	0.10	0.17
Reimbursement rate 5% more* Education_m	0.12	0.13
Reimbursement rate 5% more* Education_h	−0.05	0.17
Reimbursement rate 10% more* Education_m	0.08	0.17
Reimbursement rate 10% more* Education_h	0.05	0.21
Medium competence* Education_m	−0.11	0.14
Medium competence* Education_h	−0.17	0.18
High competence* Education_m	0.28	0.20
High competence* Education_h	0.01	0.24
Normal attitude* Education_m	0.17	0.16
Normal attitude* Education_h	0.65***	0.21
Good attitude* Education_m	0.21	0.16
Good attitude* Education_h	0.87***	0.21

*SE* standard error, *** *p* < 0.01, ** *p* < 0.05, * *p* < 0.1

## Discussion

According to the proposed plans for FDCSs, all Chinese residents will be covered by family doctor teams and receive contract services by 2020. This clearly implies that the first five-year stage of implementation will focus on establishing and refining this policy. However, to attain this goal, there is an urgent need to consider and reflect the views of the public regarding family doctor services, especially rural residents’ preferences for contract services. This is extremely important given the criticisms of the low incentives to participate in FDCSs, as demonstrated by the current low FDCS signing rate.

The key finding of this study is that rural residents highly value health care quality, including the competence and attitude of doctors, and these attributes strongly influence the uptake of FDCS contracts. This finding is in line with previous studies elsewhere. Previous studies in other settings have found that patients’ choices must be significantly influenced by the quality and experience of general practitioners [38, 39]. A previous study from China also found that the ability of primary healthcare providers was the most important factor affecting residents’ willingness to sign service contracts [18]. This suggests a major challenge for the FDCS programme, particularly in remote rural regions lacking high-quality medical resources and facing shortages of qualified primary care providers. Several studies have highlighted the poor quality of primary care services in rural areas of China. One recent study found that most general practitioners working in primary health facilities do not have college education experience and had received only 2–3 years of medical training in China [40]. Another study employing standardized patients found that general practitioners working at village clinics were able to correctly treat presumptive cases of angina 61.00% of the time and dysentery 45.00% of the time [41]. Thus, the feasibility of FDCSs may depend critically on improving the quality of primary care.

In this study, the respondents also regarded the attitude of family doctors towards patients as important. This result is also consistent with previous research that has suggested that patients valued open communication with their health care providers, underscoring a desire for good attitudes from doctors [42–44]. A good attitude can enhance patients’ trust in family doctors and promote health care continuity. Patients with regular primary care doctors have been found to be more likely to adhere to advice on treatment and prevention, improving patient satisfaction rates and decreasing hospital admissions over time [45, 46]. The preference heterogeneity analysis indicated that rural residents with higher educational attainment valued doctors’ attitudes much more than those with lower educational attainment. One possible explanation for this result is that individuals with high educational attainment have more expectations regarding kind and patient treatment. This finding is also consistent with research suggesting that a good attitude of doctors can significantly improve healthcare quality, as general practitioners can better treat patients and their healthcare issues and guide patients when trust is gained over time [45, 47].

The survey participants also considered medicine availability to be an important determinant in their FDCS selection and enrolment decision. Similar to this finding, one DCE study reported that the availability of necessary medicine at health facilities significantly impacted the probability of patients utilizing those public facilities [48]. Currently, China has a widely recognized issue of sufficient access to essential drugs at rural primary health service institutions, despite China’s national essential drug policy aiming to guarantee access [8]. Because of this lack of availability, residents who have already signed contracts with general practitioners and patients with chronic diseases often have to purchase drugs at secondary or other health facilities, undermining the effectiveness of contract services.

Monetary attributes, including the contract cost and reimbursement rate, were found to have the smallest effects. The model estimation results indicate that increasing eth contract cost would significantly decrease the willingness to sign a contract. These results are supported based on our qualitative interviews and focus group discussion. Qualitative data collected from the pilot study indicated that paying money for service contracts would lower the willingness to sign FDCS contracts for rural residents with a low household income. However, they value the outcomes and benefits that FDCSs could bring them more than the contract payment. If the services provided by general practitioners could improve their health status and make health service utilization more convenient, they would like to pay and sign a contract. Hence, policy makers should adjust the service plans to make them in line with residents’ health demand and make the cost of each service plan reasonable.

Our study had a number of limitations. Due to resource constraints, this DCE study was conducted in a single province, which may limit the generalizability of the study results to other areas in China. Regarding WTP, one previous study has suggested that the levels of the cost attribute can affect the estimates [48]. In this study, contract cost levels were determined based on a pilot study and were chosen to correspond to the existing proposed implementations of the policy. Finally, since each participant was forced to make a choice in each pair-wise choice set, the respondents may hold alternative choices and explanations not captured in our questionnaire, such as not entering into a contract with any family doctor or going only to the same family doctor. Future research should include qualitative studies to capture respondents’ choices and behaviours in a more realistic manner.

## Conclusions

This study found that rural residents valued health care quality characteristics—such as doctors’ competence, treatment skills, and attitude—more than non-quality attributes, including an increased insurance reimbursement rate, a sufficient availability of essential drugs, and the contract cost. These findings clearly suggest that policy makers must prioritize improving the quality of family doctors to increase FDCSs uptake. Specifically, policies should focus on improving the competence of family doctors, incentivizing doctors to engage in patient-centred services, and encouraging more trustful and respectful patient-provider relationships to ensure the quality of family doctor contract services. The results also suggest that uptake may be improved by offering tailored contract service packages in line with residents’ health status.

## Supplementary information

**Additional file 1.**

## Data Availability

The datasets used and/or analyzed during the current study are available from the corresponding author (Prof. Chengchao Zhou) on reasonable request.

## Abbreviations

FDCSs: Family doctor contract services
CNY: Chinese yuan
