# Supplementary material for: Analysing the preferences for family doctor contract services in rural China: a study using a discrete choice experiment
Source: BMC Fam Pract. 2020 Jul 25;21:148. doi: 10.1186/s12875-020-01223-9 (PMC7382837; doi:10.1186/s12875-020-01223-9)
Supplement: Supplementary file 1 — Additional file 1. [file 12875_2020_1223_MOESM1_ESM.docx]

**Appendix**

This appendix describes DCE methodology in terms of attributes selection and description in detail.

**DCE methodology**

The study was designed based on DCE guideline(World Health Organization 2012). and the attributes and levels selection process with description is introduced as follows.

**Attribute selection and description**

We first reviewed international and domestic literatures on primary health care providers and patients' choices of doctors to identify which attributes were highly relevant to our study. 9 attributes involving cost, waiting time, health-care quality, health facility levels, reimbursement rate, relationships between doctors and residents, treatment time, doctors' treatment technologies, and fulfillment of patients' health demand were selected at first according to literature results.

We carried out one focus group discussion which recruiting 7 key informants involving rural residents, primary physicians working at village clinics, township hospital managers. Using semi-structured interview, we collected data about what factors influence rural residents most when they sign family doctors. A DCE workshop with 2 DCE experts was also conducted in March 23-25, 2018. We invited Professor Gang Chen from Flinders University in Australia and Professor Shunping Li from Shandong University in China to discuss issues toward attributes selection. The 2 DCE experts gave valuable suggestions on attributes description, determine the levels for each attributes, and experiment designs. Finally, combining the results of literature review and qualitative researches, five attributes of family doctor contract service plans that were likely to be major considerations of rural residents in their FDCS decision-making were determined.

The attributes and levels are listed in Table 1.

**Table 1** Attributes and levels

| **Attributes** | **Levels** | **Description** |
| --- | --- | --- |
| **Cost of the contract** | 0(ref.) | Signing family doctor contract is free |
|  | 100 | Signing family doctor contract costs 100CNYper year |
|  | 200 | Signing family doctor contract costs 200CNYper year |
| **Availability of medicine** | Difficult(ref.) | It is difficult to obtain medicines from the family doctor team |
|  | Easy | It is easy to obtain medicines from the family doctor team |
| **Reimbursement rate** | Standard (ref.) | The same as medical insurance which the respondent already has. |
|  | increase 5% | The reimbursement rate will increase by 5% (based on the standard medical insurance) |
|  | increase 10% | The reimbursement rate will increase by 10% (based on the standard medical insurance) |
| **Competence of family doctor** | Low(ref.) | Family doctor always gives the wrong diagnosis or provides unnecessary treatment |
|  | Medium | Family doctor gives partially correct diagnosis and provides necessary treatment |
|  | High | Family doctor gives correct diagnosis and provides appropriate treatment |
| **Attitude of family doctor** | Poor (ref.) | Family doctor not familiar with registered residents and listens to them impatiently |
|  | Normal | Family doctor treats registered residents with respect and listens to them in a patient manner |
|  | Good | Family doctor knows registered residents well, listens to them carefully and be patient-centered |

**Table 4** Interaction effects model estimation for different attributes of contracted services.

| **Attributes and levels** | **Model 1** | **Model 2** | **Model 3** | **Model 4** |
| --- | --- | --- | --- | --- |
| Contract costs | -0.01**  (0.01) | -0.01***  (0.01) | -0.01*  (0.01) | -0.01***  (0.01) |
| Availability of medicine (easy) | 1.28***  (0.25) | 0.68***  (0.09) | 0.95**  (0.39) | 0.63***  (0.08) |
| Reimbursement rate 5% more | 0.16  (0.25) | 0.24***  (0.08) | 0.31  (0.40) | 0.35***  (0.08) |
| Reimbursement rate 10% more | 0.44  (0.30) | 0.43***  (0.10) | 0.53  (0.48) | 0.49***  (0.09) |
| Medium competence | 1.23***  (0.27) | 1.36***  (0.11) | 0.85  (0.43) | 1.33***  (0.10) |
| High competence | 2.99***  (0.40) | 2.56***  (0.17) | 2.62**  (0.59) | 2.56***  (0.15) |
| Normal attitude | 1.56***  (0.31) | 0.99***  (0.11) | 2.54***  (0.59) | 1.08***  (0.09) |
| Good attitude | 1.90***  (0.31) | 1.12***  (0.12) | 3.20***  (0.49) | 1.45***  (0.10) |
| **Interactions with demographics** | **Age** | **Sex** | **Education** | **Health** |
| Contract costs | -0.01  (0.01) | 0.01  (0.01) | -0.01  (0.01) | -0.01  (0.01) |
| Availability of medicine (easy) | -0.01***  (0.01) | -0.10  (0.12) | -0.06  (0.08) | -0.01  (0.13) |
| Reimbursement rate 5% more | 0.01  (0.01) | 0.15  (0.12) | -0.01  (0.08) | -0.11  (0.15) |
| Reimbursement rate 10% more | 0.01  (0.01) | 0.06  (0.15) | -0.01  (0.09) | -0.12  (0.13) |
| Medium competence | 0.01  (0.01) | -0.22  (0.13) | 0.08  (0.08) | -0.26*  (0.14) |
| High competence | -0.01  (0.01) | -0.22  (0.18) | -0.30  (0.09) | -0.36*  (0.18) |
| Normal attitude | -0.01*  (0.01) | 0.03  (0.15) | -0.30***  (0.09) | -0.23  (0.15) |
| Good attitude | -0.01  (0.01) | 0.01  (0.15) | -0.34***  (0.09) | -0.11  (0.15) |

Note: *** p<0.01, ** p<0.05, * p<0.1
